# Biofilm Formation and Extended-Spectrum Beta-Lactamase Producer among Acinetobacter Species Isolated in a Tertiary Care Hospital: A Descriptive Cross-sectional Study

**DOI:** 10.31729/jnma.4726

**Published:** 2019-12-31

**Authors:** Manisha Sharma, Jyotshna Sapkota, Beena Jha, Bhavesh Mishra, Chandra Prakash Bhatt

**Affiliations:** 1 Department of Microbiology, Kathmandu Medical College and Teaching Hospital, Sinamangal, Kathmandu, Nepal

**Keywords:** *biofilm*, *extended-spectrum*, *beta-lactamase*, *multidrug resistance*, *Nepal*

## Abstract

**Introduction:**

Acinetobacter species are short, stout, gram-negative coccobacilli, generally considered to be a relatively low-grade pathogen. However, its resistance towards multiple classes of antibiotics through an array of resistance mechanisms including its ability to form biofilm has led to its emergence as an important pathogen in hospital settings. This study was done to determine the prevalence of biofilm former and Extended-spectrum Beta-Lactamase producer among Acinetobacter species.

**Methods:**

A descriptive cross-sectional study was done in the clinical microbiology laboratory, Kathmandu Medical College from January to June 2019. Convenient sampling method was used. Ethical approval was taken from the Institutional Review Committee, Ref no. 2812201805. Preliminary identification followed by characterization of Acinetobacter spp. was done. Antibiotic susceptibility test was done using the Kirby-Bauer method following Clinical and Laboratory Standards Institute guidelines. Extended-spectrum Beta-Lactamase was detected by combined disc method and Biofilm detection was done using congo red agar method. Statistical Package for Social Sciences 16.0 version statistical software package was used for statistical analysis. Point estimate at 95% Confidence Interval was calculated along with frequency and proportion for binarydata.

**Results:**

Among 108 Acinetobacter species, 86 (79.7%) Acinetobacter calcoaceticus-A. baumannii (Acb) complex was seen. Seventy-eight (72%) of the isolates were multidrug-resistant, 34 (31%) of the isolates were Extended-spectrum Beta-Lactamase producer and only 10 (9.3%) of the isolates were biofilm producers.

**Conclusions:**

Multidrug-resistant Acinetobacter spp. with the ability to produce Extended-spectrum Beta-Lactamase is prevalent in our hospital settings. Strict compliance with infection control practices is necessary to curb its spread.

## INTRODUCTION

The genus Acinetobacter is a genetically diverse group of strictly aerobic, non-fermenting gram-negative coccobacilli.^1^ Acinetobacter spp. are ubiquitous in nature and can be easily obtained from soil, water, food and sewage.^2^ More than 50 species are included within the diverse Acinetobacter genus, however, the majority are nonpathogenic environmental organisms. The most common species which cause infections is A. baumannii, followed by A. calcoaceticus and A. lwoffii.^1^

Although considered to be a relatively low-grade pathogen, its resistance towards multiple classes of antibiotics through an array of resistance mechanisms including its ability to form biofilm has led to its emergence as an important pathogen in hospital settings. A. baumannii is one of the targeted pathogens in the call by the Infectious Diseases Society of America to develop new antibiotics by 2020.^3^

The aim of the study was to find out the prevalence of biofilm formation and ESBL producer among Acinetobacter spp.

## METHODS

This descriptive cross-sectional study was carried out in the clinical microbiology laboratory of Kathmandu Medical College from January 2019 to June 2019. Ethical approval was taken from the Institutional Review Committee (IRC), Ref no. 2812201805.

Convenient sampling was done and sample size was calculated using the formula;

$$\begin{array}{ll} \text{n} & ={\text{Z}}^{\text{2}}\times \text{(p}\times \text{q)}/{\text{e}}^{\text{2}}\\ & =\text{1}{\text{.96}}^{\text{2}}\times \text{0.5}\times (1-\text{0.5)}/\text{0}{\text{.1}}^{\text{2}}\\ & =96 \end{array}$$

where,

n= required sample size
p= prevalence (50%)
q= 1-p
e= margin of error, 6%
Z= 1.96 at 95 % CI

Statistical Package for Social Sciences 16.0 version statistical software package was used for statistical analysis. Point estimate at 95% Confidence Interval was calculated along with frequencyand proportion for binarydata.

Various clinical specimens including urine, sputum, aspirates, fluid, cerebrospinal fluid, pus, swab, blood, tissue, drain tip, catheter tip, endotracheal tip and CVP tip were included in the study. The labelled samples which were delivered to the microbiology laboratory in a sterile, clean, leak-proof container with no sign of contamination were evaluated. Samples without labelling, insufficient volume, inappropriate collection and transport were rejected. Based on the type of specimen, it was processed accordingly using the suitable staining technique, culture media, and biochemical tests followed by the antibiotic susceptibility test.

The non-fermentative gram-negative coccobacilli were further subjected to various biochemical reactions. The identification of the Acinetobacter spp. was done on the basis of tests like catalase and oxidase, methyl red, Voges-Proskauer, indole and urease production, citrate utilization, oxidation/fermentation tests, triple sugar iron agar test, and glucose fermentation. Chloramphenicol sensitivity test and incubation at 44°C was also performed to differentiate the species of Acinetobacter.

Antibiotic susceptibility test was performed for each isolate following the CLSI guidelines for Kirby Bauer disc diffusion technique.^5^ Phenotypic identification of ESBL producing isolates was done using disks containing cefotaxime (30μg), and cefotaxime (30μg) + clavulanic acid (10μg). Pairs of discs (cefotaxime with cefotaxime/ clavulanic acid) were placed on Muller-Hinton agar medium and ≥5mm inhibition zone of growth in cefotaxime/clavulanic than cefotaxime was regarded as ESBL producing isolate. Multidrug-resistant A. baumannii (MDR-AB) was defined as A. baumannii exhibiting resistance to three or more classes of antibiotics.^6^

Biofilm production was screened by congo red agar method.^7^ The positive result was indicated by black colonies with a dry crystalline consistency. Non-slime producers usually remained pink, though occasional darkening at the centre of the colonies was observed and this gave a bull's eye appearance. An indeterminate result was indicated by a darkening of the colonies but with the absence of a dry crystalline colonial morphology. ATCC culture of Escherichia coli 25922 and Pseudomonas aeruginosa 27853 were taken for standard reference and quality control of the test.

## RESULTS

**Figure 1:**
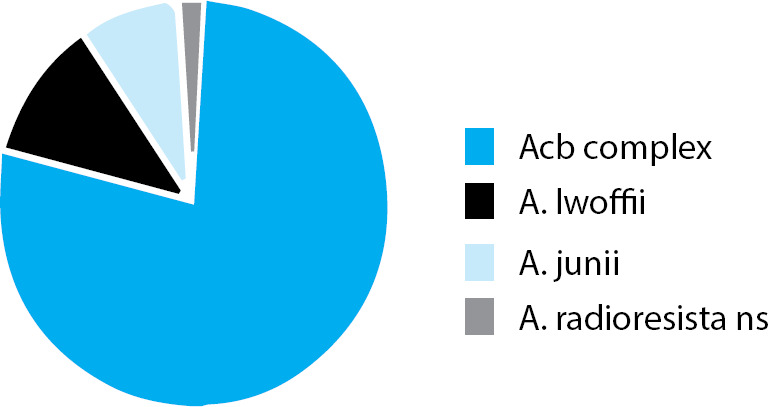
Distribution of Acinetobacter species.

A total of 108 Acinetobacter spp. isolated from various clinical specimens in the clinical microbiology laboratory, KMCTH were included in the study. Among 108 Acinetobacter isolates, the highest number of isolates were Acinetobacter calcoaceticus-A. baumannii (Acb) complex (79.7%), followed by A. lwoffii (11.2%), A. junii (8.4%) and A. radioresistans (1%) (Figure 1).

Among the various clinical specimens, the highest number of isolates was from urine (30) followed by sputum (25), tracheal aspirate (12), blood (12), pus (9), wound swab (8), device (6), Pleural fluid (2), Ascitic luid (2), Cerebrospinal fluid (1), Tissue (1).

In our study, 72% isolates were found to be Multidrugresistant. All the isolates of Acinetobacter spp. were susceptible to Tigecycline (100%) (Table 1).

**Table 1:** Antimicrobial susceptibility pattern of Acinetobacter spp.

Antibiotics used	Resistant	Sensitive
Amikacin	60.5%	39.5%
Amoxycillin/Clavulanic acid	74.2%	25.8%
Cefotaxime	95.6%	4.4%
Ceftriaxone	96.3%	3.7%
Ciprofloxacin	77.7%	22.3%
Colistin	0%	100%
Imipenem	74%	26%
Piperacillin/Tazobactam	72.8%	27.3%
Polymixin B	0	100%
Tigecycline	0	100%

Among the isolates, 34 (31%) were ESBL producers and 10 (9.3%) were biofilm producers. Among biofilm producers, 4 were also ESBL producer (Table 2).

**Table 2:** Distribution of Biofilm formers and ESBL producers.

Organism	ESBL producers	Biofilm producers	ESBL and Biofilm producers
Acinetobacter spp.	34 (31%)	10 (9.3%)	4 (3.84%)

## DISCUSSION

Acinetobacter spp. commonly cause nosocomial infections, nonetheless, community-acquired infections are also increasingly being reported.^1^ In several surveys, infections caused by Acinetobacter are primarily associated with the lower respiratory tract and the urinary tract.^1^ Similarly, in our study, among the various clinical specimen, the highest number of isolates were from urine (32.4%) followed by sputum (27%) and tracheal aspirate (12%).

The genus Acinetobacter comprises of more than 50 species of highly diverse gram-negative coccobacilli, the majority being nonpathogenic environmental organisms.^1^ Among the pathogenic, the most common species to be isolated is A. baumannii, followed by A. calcoaceticus and A. lwoffii. In our study, the most common spp. isolated was Acinetobacter calcoaceticus-A. baumannii (Acb) complex (79.7%), followed by A. lwoffii (11.2%) and A. junii (8.4%). Similar findings were reported in India and Nepal by Gupta et al, Shrestha et al respectively.^8,9^

The primary driver of clinical outcome in case of Acinetobacter infections is antibiotic resistance. In our study, the majority of the isolates demonstrated resistance to commonly used antibiotics. Maximum resistance was exhibited towards Ceftriaxone (96.3%). Resistance rates for Ceftriaxone were reported to be 91%, 93.2%, 69.3% by Shrestha et al, Joshi et al. and Amatya et al. from Nepal.^4,9,10^ These data indicate that cephalosporin might no longer be effective against Acinetobacter in Nepal.

CDC has classified Carbapenem-resistant Acinetobacter as an urgent threat in 2019 Antibiotic Resistance Threats Report.^11^ Carbapenem-resistant Acinetobacter can carry mobile genetic elements which can be easily shared between them. Some can make carbapenemase enzyme, making carbapenem antibiotics ineffective and thus effectively minimizing our armamentarium against these bacteria.^10^ Therefore, the high rate of resistance exhibited by Acinetobacter spp. towards Carbapenems (74%) in our study is alarming. Variablerate of Carbapenem-resistant Acinetobacter (17%, 47.3%, 97.7%) has been reported from Nepal.^4,9,10^ The data may reflect the haphazard usage of carbapenems and inadequate infection control practices in some of the settings driving the selection pressure among the organism and dissemination of these resistant strains in those settings.

Interestingly, we found Amikacin to be more effective than carbapenem (39.6% vs 27.6%) in our study. This may be due to increased use of carbapenems in intensive care settings for the treatment of MDR pathogens.

72% of the isolates in our study were MDR. Similar findings were reported by Amatya et al. (71.3%) and Thapa et al (82.7%).^4,12^ MDR Acb severely limits the treatment choices available to the clinician and is also associated with increased morbidity and mortality.

In our study, none of the isolates was resistant to Tigecycline and Colistin.

In our study, 31% Acinetobacter spp. were ESBL producers. Similar rates have been reported by Gupta et al, Singh et al (31.5% and 27.5%) respectively. Different studies from Nepal have reported the rate of ESBL production in Acinetobacter spp. to be 15.87%, 15.4 % by Dumaru et al. and Parajuli et al.^13,14^

Infections with Acinetobacter spp. are associated with mechanical ventilation, catheterization, surgical and invasive procedures and in patients receiving broadspectrum antimicrobials for a long duration.^1^ Factors responsible for this include colonization and selection pressure. Similarly, Acinetobacter is also capable of forming biofilm on abiotic surfaces which mediates its colonization of hospital equipment and indwelling devices such as catheters, Endotracheal tubes.^15^ In the current study, 10 (9.3%) of the isolates were biofilm producers by Congo red agar method. In a similar study in Nepal, 10% Acinetobacter spp produced biofilm.^16^ However, a study by Dumaru et al. has demonstrated a high level of biofilm production in Acinetobacter spp (53.97%).^13^ CRA method seems to be a less sensitive method for detection of biofilm than microtiter plate assay, tube adherence method, tissue culture plate method, etc, which might explain the low level of biofilm formation detected in our study. Furthermore, biofilm formation in Acinetobacter is dependent on several factors like the presence of antibiotic resistance genes, growth conditions and cell density. Molecular studies and Biofilm detection using multiple methods could not be done due to limited resources.

## CONCLUSIONS

High prevalence of Multidrug-resistant and Carbapenem-resistant Acinetobacter spp. in our settings is a cause of concern. CDC 2019 report has escalated Carbapenem-resistant Acinetobacter to Urgent because of the emergence of easily spread resistance in Acinetobacter and the lack of current and developing antibiotics to treat these infections. Therefore, addressing this problem requires preventing infection, better antibiotic usage and controlling the dissemination of infection. This can be achieved by better compliance with hospital infection control practices and effective antibiotic stewardship program.

## Conflict of Interest

**None.**
